# A Survey of Localization Methods for Autonomous Vehicles in Highway Scenarios

**DOI:** 10.3390/s22010247

**Published:** 2021-12-30

**Authors:** Johann Laconte, Abderrahim Kasmi, Romuald Aufrère, Maxime Vaidis, Roland Chapuis

**Affiliations:** 1 Clermont Auvergne INP, CNRS, Institut Pascal, Université Clermont Auvergne, F-63000 Clermont-Ferrand, France; abderrahim.kasmi@etu.uca.fr (A.K.); romuald.aufrere@uca.fr (R.A.); roland.chapuis@uca.fr (R.C.); 2 Northern Robotics Laboratory, Université Laval, Quebec, QC G1V 0A6, Canada; maxime.vaidis.1@ulaval.ca

**Keywords:** survey, autonomous vehicles, localization, intelligent transportation systems

## Abstract

In the context of autonomous vehicles on highways, one of the first and most important tasks is to localize the vehicle on the road. For this purpose, the vehicle needs to be able to take into account the information from several sensors and fuse them with data coming from road maps. The localization problem on highways can be distilled into three main components. The first one consists of inferring on which road the vehicle is currently traveling. Indeed, Global Navigation Satellite Systems are not precise enough to deduce this information by themselves, and thus a filtering step is needed. The second component consists of estimating the vehicle’s position in its lane. Finally, the third and last one aims at assessing on which lane the vehicle is currently driving. These two last components are mandatory for safe driving as actions such as overtaking a vehicle require precise information about the current localization of the vehicle. In this survey, we introduce a taxonomy of the localization methods for autonomous vehicles in highway scenarios. We present each main component of the localization process, and discuss the advantages and drawbacks of the associated state-of-the-art methods.

## 1. Introduction

Since the last decade, autonomous vehicles start to roam the road alongside human drivers on highways. In the context of Advanced Driver-Assistance Systems (ADAS), a fundamental aspect of a fully autonomous vehicle is its ability to properly perceive its environment and localize itself on the road. As pointed out by Bresson et al. [1] in their survey on Simultaneous Localization And Mapping (SLAM) algorithms, the use of such algorithms is challenging for autonomous vehicles in outdoor environments. In the context of highways, the very high velocity of the vehicle do not allow standard SLAM algorithms to perform well. Indeed, rapid motions severely hamper these techniques, as the lidar would not be able to capture enough overlapping features and the cameras would be significantly affected by the blur resulting from the speed. Intelligent vehicles must therefore rely on the available information, namely the road markings as well as the surrounding vehicles. In addition, in most applications, not only the position of the vehicle is required, but the number of lanes and the road on which the vehicle is traveling are often valuable information. Nevertheless, SLAM algorithms do not give such data even if this information is available in the environment. In light of these considerations, localization algorithms meeting these constraints have been designed, relying heavily on the structural properties of highway environments.

In this survey, we present and compare the state-of-the-art methods of localization methods for ADAS in highway scenarios. For surveys about more generic urban localization, the reader can refer to Kuutti et al. [2], Elhousni and Huang [3], Badue et al. [4].

The localization aspect on highways can be distilled in smaller components, that are

**Road Level Localization (RLL)**: The road on which the vehicle travels;
**Ego-Lane Level Localization (ELL)**: The position of the vehicle in the lane in terms of lateral and longitudinal position; and
**Lane-Level Localization (LLL)**: The position of the host lane within the road (i.e., the lane on which the vehicle travels).

For the RLL, digital maps (e.g., Google, OpenSteetMap (OSM), Waze) are used to perform this task. Global Navigation Satellite Systems (GNSS) receivers are used to retrieve the geographic coordinates (i.e., latitude, longitude, and altitude), and a Map-Matching procedure is performed to match the position of the ego-vehicle with the correct road. However, the accuracy of the localization obtained is in the order of meters. Indeed, according to the Federal Aviation Administration GPS Performance Analysis Report [5], the accuracy of a standard GPS device is within 3 m with a 95% confidence, which is not sufficient for most ADAS that require a more precise localization.

For some applications like lane-keeping, knowing the road on which the vehicle is traveling is not sufficient. These systems must be informed about the position of the host lane in the road to provide adequate maneuver instructions and maintain the vehicle’s safety. Furthermore, autonomous vehicle applications need a more accurate localization, which can be translated by the knowledge of the lateral and longitudinal positions of the vehicle in the ego-lane. For instance, overtaking maneuvers need a faultless knowledge of the lateral position of the ego-vehicle with respect to the ego-lane marking in order to decide whether the vehicle should overtake the obstacle or not. The task of vehicle localization is still challenging for an autonomous vehicle, as a complete ego-vehicle localization framework must perform all the three key aforementioned components.

We present sequentially the three identified components that an autonomous vehicle requires to safely localize itself in a highway environment, that are the Road Level Localization (RLL), Ego-Lane Level Localization (ELL) and Lane-Level Localization (LLL) components. For each component, we present the most performing methods at the time of the writing of this survey and discuss their main advantages and drawbacks.

## 2. Road Level Localization (RLL)

The ubiquity of positioning devices on the vehicles allows the drivers to know the vehicle’s position in the world. However, this estimation is very noisy due to the inherent inaccuracy of these positioning devices. To address this problem, a correcting procedure is required, that matches the vehicle position with a road network coming from a map. This technique is called Map-Matching. As stated by Quddus et al. [6], Map-Matching not only enables the physical location of the vehicle to be identified, but also improves the positioning accuracy if good spatial road network data are available. This means that Road Level Localization knowledge is determined by Map-Matching algorithm. The RLL is also a prerequisite component of several applications. An exhaustive list of applications is available in [7]. This section details and classify the state of the art on the Road Level Localization (RLL) techniques.

### 2.1. Terminologies

Before presenting the Map-Matching techniques, we start by formalizing the Map-Matching problematic. The Map-Matching problematic have been studied over two decades, and thus several formalizations have been proposed. In the following, the definition presented inherits from the one presented in [8].

**Definition** **1.** *A trajectory $T_{r}$ is a sequence of chronologically ordered spatial points $T_{r}=$$\left\{p_{1},p_{2},\cdots ,p_{n}\right\}$ sampled from a continuously moving object. Each point $p_{i}$ consists of a 2-dimensional coordinate $\left(x_{i},y_{i}\right)$, a timestamp $t_{i}$, a velocity $v_{i}$ (optional) and a heading $\theta_{i}$ (optional), namely $p_{i}=\left\{x_{i},y_{i},t_{i},v_{i},\theta_{i}\right\}$.*

**Definition** **2.** *A map is a directed graph G=(V,E), in which each vertex (x,y)∈V represents an intersection or a road end, and each edge (s,e,l)∈E is a directed road starting from vertices s to the vertex e, connected by a path l represented by a sequence of consecutive segments (polyline).*

**Definition** **3.** *The Map-Matching is a procedure that finds a route MR(Tr) for a given map G(V,E) and a trajectory $T_{r}$. The route $\mathrm{MR}(T_{r})$ represents the sequence of roads traveled by the trajectory.*

### 2.2. Online Map-Matching

Due to the importance of the Road Level Localization (RLL), Map-Matching has been subject of ongoing research since the emergence of Global Positioning System (GPS) in the 1990s [9]. According to the literature, Map-Matching techniques can be divided into two categories, namely, online and offline modes. In online mode, the Map-Matching procedure is performed in a streaming fashion, meaning that for each point $p_{i}$, a Map-Matching is performed. Consequently, the procedure has to be adequate for real-time applications. In contrast, offline Map-Matching waits until the trajectory $T_{r}$ is completed in order to perform the Map-Matching on its entirety. Hence, this procedure is not concerned about the real-time requirements. In this survey, we will focus on online Map-Matching as localizing the autonomous vehicle at each sample time is the most common use in intelligent transportation systems. However, one can note that the majority of the techniques which will be presented can be used in both online and offline modes.

**Figure 1:**
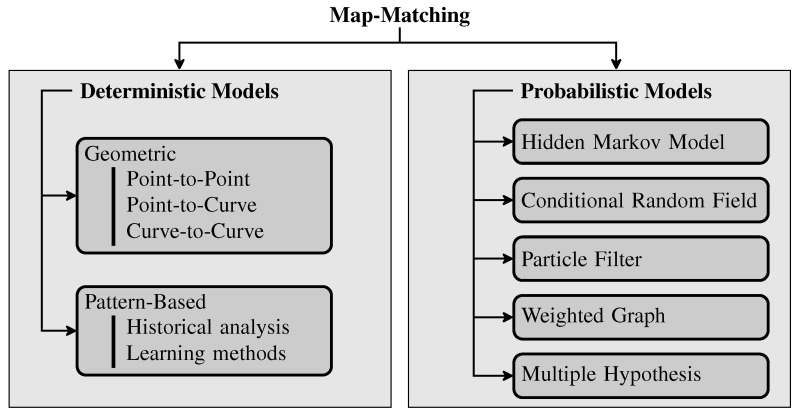
Map-Matching classification, splitting the techniques into two main parts that are the deterministic and probabilistic approaches.

Considering online Map-Matching methods, the most complete and cited survey about the subject was presented by Quddus et al. [6], followed by Kubicka et al. [9]. The authors classified the Map-Matching techniques from a methodological perspective into four categories, namely geometric, topology, probabilistic and advanced. However, after several years of research on Map-Matching methods, most of the methods mentioned in these surveys have been outperformed and new technologies have emerged, rendering the classification out of date. With a view to bringing up-to-date Map-Matching techniques, Kubicka et al. [9] proposed a survey that classifies the Map-Matching methods depending on the application. To the best of our knowledge, there is still no consensus on how to classify the Map-Matching methods. However, from our point of view, the classification presented in the survey [8] is the most up-to-date and accomplished. Indeed, it summarizes most of the existing solutions and provides guidance to future research. Therefore, we propose to enhance the proposed classification in [8]. We classify the existing Map-Matching methods into two different classes, namely (1) Deterministic Models and (2) Probabilistic Models. Each class is itself decomposed in subclasses. In the following, we will detail each category depicted in Figure 1.

### 2.3. Deterministic Model Approaches

In these approaches, the Map-Matching returns the link that is the closest to the trajectory geometrically or topologically. In a perfect world, the vehicle’s trajectory matches the closest road network topology. Hence, in this model, the main focus is on how to define the closeness between the trajectory and the road.

#### 2.3.1. Geometric Algorithms

The geometric algorithms are the most commonly used and oldest methods [10]. These methods were introduced in 1996 by Bernstein et al. [11]: the authors denominate the methods as point-to-point, point-to-curve, or curve-to-curve. The most elementary approach, the so-called point-to-point, matches each position sample to the nearest node in the map. The point-to-curve approach projects each position sample to the geometric-closest road. Lastly, curve-to-curve methods match the vehicle’s trajectory $T_{r}$ to the geometric-closest link in the road-network. In [10], the authors compared four basic Map-Matching methods based on geometric algorithms. The first one is the classical point-to-curve with no consideration of the vehicle state or the road-network. The second one is a modified version of the point-to-curve: in this version, the vehicle’s heading was taken into account in the matching process. The third method is an upgrade of the second one, taking into consideration the topology of the road network. The last one is the curve-to-curve method as presented before. The experiment was conducted on four routes that were traveled in the town of Mercer County in New Jersey. None of these routes involved highways or arterials. The results have been summarized and reported in Table 1. The matching accuracy correspond to the proportion of correctly matched samples over the whole dataset. As expected, methods taking into account more context, e.g., the heading or the route contiguity, lead to better results.

All the mentioned methods follow the same paradigm. Indeed, the differentiation appears in the definition of the closeness. In the literature, similarity metrics earned a lot of attention. Quddus and Washington [12] proposed a compound metric by fusing several distances, such as the distance from the curve and the difference of heading directions. Nonetheless, the most popular metric is the Fréchet distance. The Fréchet distance was first defined in [13]. This distance can be illustrated by the following example: a person is walking on a certain curve, and a dog is walking on another one. We assume that both have free control over their speeds but are not allowed to go backward. In this example, the Fréchet distance of the curves is the minimal length of a leash between the person and the dog that is required to cover the curves from start to finish. Mathematically, the Fréchet distance was defined as follows [13]:

(1) $$\delta_{F}\left(f,g\right)=\underset{\alpha ,\beta }{\inf}\max_{t\in [0,1]}∥f\left(\alpha (t)\right)-g\left(\beta (t)\right)∥,$$

where ∥·∥ is the standard euclidian distance, f,g are two parametric curves, and α (respectively β) is a continuous, monotonic, increasing reparametrization from the domain of *f* (respectively *g*) to [0,1]. Alt et al. [14] pioneered the Fréchet distance for Map-Matching. They were able to find a route whose Fréchet distance to the trajectory is minimal. However, they pointed out one of the major counterparts of the distance: as the distance relies on the maximum distance between the two curves, any outlier in the trajectory would lead to a wrong estimation. Following this work, Brakatsoulas et al. [15] presented a relaxed version of the Fréchet distance called the weak Fréchet distance, lifting assumptions on the reparametrizations α and β. Using the weak Fréchet distance, the authors were able to lower the computational requirements. In the same context, further works have been presented with the objective of speeding up the Fréchet distance [16,17,18].

#### 2.3.2. Pattern-Based Algorithms

Pattern-based methods are well-known in the literature. The assumption is that given a start and an endpoint, people tend to travel on the same trajectory [19]. In that sense, giving a pair of a start and endpoint, and taking into account historical Map-Matching results, the method will find the most similar trajectories that the vehicle will travel on. Finally, the algorithm will decide on the optimal route based on a scoring function. Recently, Li et al. [20] were the first ones to propose a deep learning approach that is able to create representations of trajectories. The objective is to capture the route information of each trajectory. In the same manner, Zhao et al. [21] presented DeepMM: a deep learning Map-Matching system. The main drawback of the pattern-based algorithms is the sparsity and disparity of the historical data. In that sense, the historical data may not cover all the new queried trajectories, which can lead to false Map-Matching results.

### 2.4. Probabilistic Model Approaches

Although the position data is necessary, it cannot be taken as the sole predictor of the vehicle’s path. Indeed, naively matching this noisy path to the nearest road using deterministic metrics presented in Section 2.3 will eventually result in irrational paths involving counterintuitive driving behaviors. Hence, a Map-Matching algorithm has to consider the reasonableness of a given path in relation to the vehicle dynamics. In that sense, the Map-Matching algorithms presented in this section share the same paradigm, which is the probabilistic reasoning, whether it is for the reasonableness of the path, or for the vehicle’s dynamic state.

#### 2.4.1. Hidden Markov Model (HMM)

Hidden Markov Model (HMM) models for Map-Matching have been the subject of numerous research studies in connection with tracking problems. The architecture of the Map-Matching made it suitable to model the road network topology. The enthusiasm for the HMM in the Map-Matching problematic was initiated by Hummel [22], resulting in dozens of methods using Map-Matching. Most of them were designed for offline Map-Matching. However, as claimed by Newson and Krumm [23], online Map-Matching is possible using the sliding window technique. Basically, the aim of the framework is to find the most likely road given a trajectory $T_{r}=\left\{p_{1},\ldots ,p_{n}\right\}$. To perform such a task, the standard method is based on the Viterbi algorithm [24]. The algorithm runs on $O\left(nm^{2}\right)$, with *n* the number of observations (i.e., the vehicle’s estimated positions $p_{i}$) and *m* being the number of states (i.e.,  the possible roads $l_{j}$). As said earlier, using the sliding window technique can reduce the time complexity. Therefore, the majority of the studies do not differ in the architecture or the representation of the Map-Matching task using the HMM. Nonetheless, they do differ in the definition of the emission probability and the transition probability. Historically, HMMs were first used by Hummel [22] for offline Map-Matching. The transition probabilities were uniformly distributed while taking into account the turn restriction. The method is tributary to the vehicle’s heading estimation, which is estimated by taking into account two consecutive GNSS measurements. In the case of Map-Matching with inaccurate GNSS data, this can be pathological. In particular, when the distance between two GPS measurements is small, the errors on the vehicle’s heading can be considerable [9]. The method was later extended by Pink and Hummel [25], resulting in a more robust method. A Kalman Filter was introduced to filter the initial trajectory obtained in order to eliminate outliers. However, the most important contribution was the structuring of the road network using cubic spline interpolation instead of linear interpolation. The motivation behind this modeling is to make the method more robust against the heading errors, which was the main issue with the previous method [22]. Besides, it better reflects the trajectory shapes that vehicles normally follow. Following, instead of relying on the vehicle’s heading estimation, Newson and Krumm [23] proposed a HMM in which the transition probability is governed by a “route distance”. The assumption made by the authors is that the correct route has the smallest difference between two consecutive estimated vehicle positions with the distance along the road-network. The same method was adapted and extended later by Luo et al. [26]. Jagadeesh and Srikanthan [27] enhanced a HMM with the concept of “drivers’ route choice”. The authors use a route choice framework that models the fact that the driver have preferences for certain paths compared to others (e.g., a shorter path is always preferred). Finally, Kasmi et al. [28] proposed to use a low precision GNSS sensor to perform Map-Matching, relying on several probabilistic criteria. They enhanced their work in [29] by using a HMM based on [26] to model the time-dependency of the road estimation.

The HMM-based Map-Matching achieved an accuracy comparable to the state-of-the-art geometric models [18]. However, HMM based methods suffer from mainly two main drawbacks. The first one is the selection bias problem, as pointed out by Hunter et al. [30], which is a side effect of the HMM when giving more weights to long disconnected segments of road. For instance, it is particularly troublesome in the case where a highway is close to a network of smaller roads: the HMM will give a higher weight to the highway and considerably smaller weights to the road network because of the transitions between the smaller paths and their possible large number, decreasing each other probabilities. As such, the highway may be preferred by the HMM even if the vehicle is closer to the road network. The second one is that these methods are not robust against missing trajectory samples. Indeed, the structure of the transition model in a HMM takes into account the connectivity, physical, and logic between two consecutive sets of route candidates. The discontinuity in position frames will jeopardize the travel possibility between these route candidates. To overcome these issues, more sophisticated methods have been developed and are presented below.

#### 2.4.2. Conditional Random Field (CRF)

The Conditional Random Field (CRF), unlike HMM, is a probabilistic framework that is not restricted by the Markov independence assumption. In theory, CRF models higher-order interactions between more than two states. In other words, it can model the interactions between the observation at the current state and its predecessor. That is from a theoretical point of view, as in the literature, the existing CRF are confined to the first-order dependencies between adjacent states. Hunter et al. [30] introduced a CRF for Map-Matching as an alternative to the classical HMM. The overall sophisticated methods model the spatial and temporal relationship as well as the classical HMM. Furthermore, the authors integrated the driving behavior in addition to the vehicle speed. Using the same paradigm, Yang et al. [31] characterize a CRF model for Map-Matching. To verify the effectiveness of the model, the authors performed the Map-Matching on a dataset from Shanghai taxis. Even if the overall accuracy reached was significant, the CRF used considered only first-order dependencies between states. Therefore, the CRF share the same inability as the HMMs to take into account contextual information. In addition, a learning procedure is required for the CRF to model the interactions between these states, which makes the CRF easy to utilize but heavy to structure.

#### 2.4.3. Weighted Graph Technique

More sophisticated techniques have been developed to take into account the spatial geometric and topological structures of the road network, in addition to the temporal and speed constraints. One of these techniques is referred as Weighted Graph Technique [8]. The matching process is performed through a weighted candidate graph. Lou et al. [32] introduced the st-matching algorithm, in which the Weighted Graph Technique process is summarized as three steps: (1) Candidate Preparation: In this step, the candidate graph is initialized. Similar to most Map-Matching techniques, the candidates are selected based on a radius of measurements from the estimated position (GNSS position). (2) Spatial and Temporal Analysis: This step is composed of two components. First, similar to HMM-based method, an observation probability and a transition probability are emitted to each candidate. These two probabilities are inferred from a scoring function that takes into account the distance between the position and the candidate, in addition to the road topology. The second component is a temporal analysis in which the speed of the vehicle is compared with the typical speed constraints on each candidate path. The objective of the spatial and temporal analysis is to weigh edges in the graph. (3) Result Matching: In this last step, the path is inferred based on the constructed weighted graph.

Globally, methods that fall into this category share the same design as the one presented by Lou et al. [32]. They only differ in the scoring function in the spatial and temporal analysis. Hu et al. [33] consider more sophisticated parameters. Authors took into consideration the reciprocal effects between adjacent candidates, the reasonableness of travel time, and other road characteristics such as traffic lights.

#### 2.4.4. Particle Filter

Instead of dealing with an estimated position as an independent element, some researchers focus their attention on the trajectory of the vehicle. The idea is to filter the trajectory by coupling internal information from the microelectromechanical device (MEMS) such as gyroscopes and accelerometers, with GNSS. In essence, there exist two types of filters, namely the linear and non-linear. For the linear filters, errors due to the imperfection of the model and sensors are represented by Gaussian white noises and are linearized using first order Taylor approximations. Based on the assumption of additive Gaussian white noises, the estimation of these error states can be obtained with an Extended Kalman Filter, for instance. In contrast, a non-linear filter does not require linearization, therefore, from a theoretical point of view, no errors result from such a step. In the context of Map-Matching, Dmitriev et al. [34] acknowledged that during a vehicle turn, the posterior distribution of the vehicle position on the road is non-Gaussian. Because of that, non-linear filtering methods are required to solve this problematic. Tackling this non-linear problematic, Particle Filter-based methods are used. Initially, the Particle Filter has been used as support prior to the Map-Matching process, by fusing sensors information to estimate the vehicle’s state. For example, Toledo-Moreo et al. [35] proposed a Lane-level localization Map-Matching where he introduced a Particle Filter to fuse sensors information in order to estimate the vehicle a priori position. In general, the Particle Filter is structured as follows. In the initial phase, $N_{p}$ particles are sampled. These particles represent the different hypothesis of the vehicle’s localization, and they all receive the same weight. For each particle, its associated weight is updated accordingly to its likelihood of existence, as soon as a new observation is received. Afterward, a resampling stage starts: particles with low weights are likely to be erased, and the ones with higher weights are used in a vehicle cinematic model in order to feed particles of the next cycle. Therefore, all the methods that fall into this category share the same strategy [36], they only differ in the definition of the weighting function for the particles. However, one major drawback of these methods is that they employ a vehicle dynamical model that does not work for data that have a low sampling rate (i.e., a rate of the order of magnitude of several seconds).

#### 2.4.5. Multiple Hypothesis Technique

Historically, Multiple Hypothesis Technique methods were aimed for military tracking aircraft like airplanes and missiles. Indeed, in 1979, Donald B. Reid, a U.S. Military engineer, developed a tracking multiple targets and multiple hypothesis algorithm [37]. The algorithm was adapted by Pyo et al. [38] into single target tracking with multiple hypotheses. The authors demonstrated that although the initial algorithm was designed for aircraft, it can easily be adapted to Map-Matching. The authors proposed a Multiple Hypothesis Technique in which each hypothesis is associated with a probability.

The Multiple Hypothesis Technique, as the name suggests, holds a set of candidates or hypotheses during Map-Matching. The set of hypotheses is generally initialized based on a simple geometric metric. Afterward, the set of hypotheses keeps evolving as further observations are received. According to Kubicka et al. [9], the evolving process consists of two processes, namely, hypothesis branching and hypothesis pruning. A hypothesis is branched or replaced when the vehicle travels the candidate and therefore arrives at a crossroad. The original parent hypothesis is then replaced by new child hypotheses. The new child hypotheses are an extension of the parent hypothesis by taking into account all the directions that the vehicle can take at the crossroad, which guarantees that there will be at least one hypothesis covering the correct candidate in which the vehicle will travel. Another advantage of the method is that some failures are intuitively spotted. If there are no hypotheses, it necessarily implies that a problem has occurred at some points. Hypothesis pruning consists of the elimination of the unrealistic hypothesis. The process is based on a pruning criterion: in the state-of-the-art methods, this pruning criteria differs from one author to another. However, the main idea is to model criteria that allow to keep the most likely hypothesis and simultaneously eliminate the most unlikely hypothesis.

The pruning strategy consists of eliminating the hypothesis for which the probability goes under a certain defined threshold. The authors also considered the hypothesis with the highest probability and compared it to a predefined threshold to know if the hypothesis is confirmed. The presented method showed significant results with a range of 4% to 17% of a miss-match. However, this method is not optimal in the sense that optimal Map-Matching technique does not require any hand-tuning. Continuing on the same paradigm, Marchal et al. [39] and Kubička et al. [40] proposed their own version of Multiple Hypothesis Technique. The contributions are essentially based on making the framework simpler and faster. In addition, Kubička et al. [40] presented a framework that does not require access to the vehicle odometry and gyroscope data. Compared to HMM models, Multiple Hypothesis Techniques are more robust to mismatches since the current Map-Matching is not governed by transition state that is related to the previous solution. However, in worst-case scenarios, the set of hypotheses can grow exponentially, and therefore, the pruning process is critical. The pruning strategies have to be robust to outlier hypothesis, but, at the same time, have to be flexible in order not to eliminate the hypotheses that are likely to be correct. On the one hand, the pruning process is more prone to errors, if not treated properly. On the other hand, there exists no formal proof for pruning strategies, or criteria, that ensure this condition. To overcome this issue, Quddus [41] introduced the notion of “integrity monitoring”, which was inherited from aerial navigation where it was used to verify the reliability of critical aerial navigation systems like satellites and missiles. In the context of Map-Matching, the need for “integrity monitoring” emerges from the fact that correct Map-Matching is not always possible when there are some strong ambiguities and some incongruities between the path and the map. In these situations, it is necessary to report the reliability of the Map-Matching output to the user. To this end, Jabbour et al. [42] followed by Li et al. [43] proposed different metrics to ensure that the output of the Map-Matching is coherent, and more importantly, to report false Map-Matching results. Jabbour et al. [42] proposed to monitor the coherence of the system by checking that the sum of the weights (i.e., likelihoods) of the hypothesis remains above a certain threshold, ensuring that some hypotheses are rightfully following the true state of the vehicle. Li et al. [43] chose to control the integrity of the estimation by tracking the innovation in the filtering process. In the case that a fault occurs in the system, the innovation will be greater than usual and such a technique will detect the fault and raise an alarm.

### 2.5. Conclusion of Road Level Localization

In the light of the investigated literature, we discuss what are the main requirements that need to be necessarily addressed by a Map-Matching algorithm. Despite more than twenty years of development on Map-Matching, there is not yet a solution that deals with all scenarios. To the best of our knowledge, there exists no consensus on how Map-Matching methods should be rated. Indeed, the majority of the authors utilize their own dataset. Nevertheless, according to the literature reviews [8,9], the majority of the Map-Matching algorithm reaches an accuracy of around 95%. Accordingly, the accuracy of the Map-Matching will not be discussed in details. Indeed, the major bottleneck in Map-Matching algorithms development is the absence of a unanimous agreement on one publicly accepted dataset. For that reason, the comparison between algorithms is still an open problem. With that background, we propose a different type of comparison, taking into account the most essential aspects of a Map-Matching that are uncertainty-proof, matching break, integrity indicator, and run time.

- Uncertainty-Proof is the ability of the Map-Matching algorithm to take into account inherent uncertainties that come from the raw data;
- Matching Break describes the capability of the Map-Matching algorithm to propose a solution where there is a break in the GNSS data;
- Integrity Indicator is a trust indicator on the validity of the output of the Map-Matching algorithm, which can be relevant for the ambiguous cases; and
- Run Time of the frameworks: in order to be used in an autonomous vehicle, the Map-Matching algorithm has to fulfill real-time requirements.

Based on these criteria, we rated each method based on the previous analysis, and we reported the evaluation in Table 2. The notation ranges from −− to ++, respectively being the worst and best notations.

On the one hand, deterministic methods are straightforward techniques that do not require complex computation. As a result, the running time of the algorithms is very low compared to the other methods. However, these methods are very tributary to the quality of the raw data used. Therefore, they suffer from the incapability to handle uncertainties and ambiguities. On the other hand, probabilistic methods are analytically different from each other. They are capable to handle uncertainties and matching break situations. The backlash is that they are complex and require more computations. In that sense, a hybrid architecture including a preprocessing step based on a deterministic method and a selection stage based on a probabilistic method has the utility of dealing with the limitations raised from each one of the methods. Indeed, the processing stage is straightforward and does not necessitate extra computations. Therefore, it has the strength to deal with the majority of the nominal case. On the other side, the ambiguous cases can be solved using a probabilistic method, which has the ability to deal with these situations. By doing so, the complexity of the Map-Matching problematic is reduced. We believe that finding the optimal equilibrium between deterministic and probabilistic approaches will yield a powerful Map-Matching algorithm. In the next sections, we investigate local localization methods which constitute two of the three main components of a localization architecture.

## 3. Ego-Lane Level Localization (ELL)

For some applications like lane-keeping, being aware of the road on which the vehicle is traveling is not sufficient. These systems must be informed about the position of the host lane in the road to provide adequate maneuver instructions and maintain the vehicle safety. Furthermore, autonomous vehicle applications need a more accurate localization which can be translated by the knowledge of the lateral and longitudinal positions of the vehicle in the ego-lane. For instance, overtaking maneuvers need a faultless knowledge of the lateral position of the ego-vehicle with respect to the ego-lane marking in order to decide whether the vehicle should overtake the obstacle or not.

McCall and Trivedi [44], Zhu et al. [45] summarized and illustrated the main objectives of lane position-detection algorithm systems in their researches. The characteristics of these systems are distilled as follows:

- Lane-Departure-Warning Systems: It is essential to accurately estimate the position of the vehicle with respect to the ego-lane marking.
- Adaptive Cruise Control: Measures such as the smoothness of the lane are crucial for this monitoring work.
- Lane Keeping or centering: The aim is to keep or center the vehicle in its host lane. As a result, a faultless estimation of the lateral position is required (e.g., [46]).
- Lane Change Assist: It is mandatory to know the position of the ego-vehicle in its host lane. The lane change has to be done without any risk of colliding with an obstacle (e.g., [47]).

Under these considerations, we propose in this section a classification of the algorithmic techniques independently on the various modalities used (e.g., camera, lidar, radar) in order to detect the ego-lane marking.

**Figure 2:**
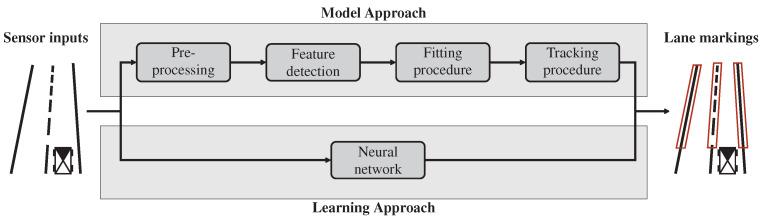
Classification of the algorithms used for ego-lane marking detection. Model approaches dissect the problem into independent submodules, whereas learning approaches are based on end-to-end methods.

Leafing through the literature, it appears that most researchers use lane marking detection to provide an accurate ego-lane level localization. Lane marking detection has been an active field of research for the past three decades and great progress has been made in the past few years. It is possible to categorize existing approaches to lane marking detection into modular pipelines, or model-driven and monolithic end-to-end learning approaches. As shown in Figure 2, both approaches are juxtaposed at a conceptual level. The standard approach to lane marking detection is the model-driven approach. The main concept is to break down the lane marking detection into modules that can be independently developed and tested. Modular pipelines have the main advantage of deploying human-interpretable intermediate representations to understand system failure modes. A significant drawback to modular methods is that intermediate representations built by humans are not inherently suitable for tasks such as the identification of lane markers. An alternative to modular pipelines is end-to-end learning-based models based on neural networks. The network parameters can be learned via a training dataset or using a learning transfer technique from trained networks. These approaches reach significant accuracy in several computer science domains. However, most networks are trained and validated on one dataset, which makes the network less generalized to other datasets. Moreover, as claimed by Janai et al. [48] neural network-based approaches are often hard to interpret as they present themselves as “black boxes” to the user which does not reveal why a certain error has occurred.

The survey presented by Hillel et al. [49] focused on the lane marking detection. The authors presented a review of ongoing research on road and lane detection, and covers a large part of the lane marking techniques used at that time. In addition, the authors presented the techniques without differentiating on the sensor used. Nonetheless, this study was published in 2014, at a time when the prevalence of neural networks was not as pronounced as it is now. Therefore, their study presented only model-driven approaches without learning ones. Concerning the learning approaches, there has been an considerable amount of effort invested in deep learning techniques for autonomous vehicles and especially for the perception task. In that sense, the survey presented by Fayyad et al. [50] covers most of the recent deep learning techniques. Contrary to the study presented by Hillel et al. [49] and other works presented in [44,51], the focus and the ambition of this section are to emphasize the recent studies that dealt with lane marking detection, which included deep learning algorithms. The first part of the section is dedicated to the main existing model-driven approaches. Then, the second part will present the learning approaches used for the ELL. Finally, this section concludes by highlighting and summarizing some current trends approaches in lane marking detection, and a comparison between the methods is given.

### 3.1. Model-Driven Approaches

Inspection of model-driven lane marking detection literature brings to light that most approaches share the same functional architecture. Therefore, we depict the commonalities between all the encountered algorithms into a generic system, whose components are divided into four steps, namely, a pre-processing step, succeeded by a road marking feature detection, then a fitting procedure, and finally a tracking procedure. An illustration of these components are illustrated in Figure 2 (top). We use this generic system as a skeleton, enabling comparison between different algorithms according to their functional parts. Naturally, feedback connections also exist between higher modules (e.g., fitting procedure) that guide lower module (e.g., pre-processing).

#### 3.1.1. Pre-Processing

In general, a set of several operations that can be applied to a frame input before feature extraction is called pre-processing. The frame used is commonly called the world frame, which embraces both lidar data and camera image. This pre-processing block is generally the first process of lane marking detection. The objectives of pre-processing are to enhance features of interest, reduce clutter, and remove misleading artifacts. Thereafter, the cleaned image is used for feature extraction. According to Hillel et al. [49], the methods that fall under this module’s scope can be clustered into two classes: handling of lighting-related effects, and pruning of non-relevant or misleading parts of the image.

A robust lane marking should be capable of handling different lighting conditions, which are constantly changing because of the effects of the time of day and weather conditions. These lighting conditions can vary from sunny midday to nighttime artificial illumination. In addition to these natural changes, the lane marking solution might be confronted with a drastic lighting switch when entering or exiting a tunnel, or while being under a bridge that covers the sunlight. Another illumination phenomenon that has to be brought seriously to the table is the illumination of the lens flare caused by the sun’s rays hitting the camera’s field of view. Huang et al. [52] used the “absolute calibration” of the camera to allow the computation of solar ephemeris, which allows deducing the sun location on the image and hence suppress the line estimations that point toward the sun location on the image.

Furthermore, a major source of clutter is the shadows cast on the surface of the road. Their edge intensity can be ambiguous for some gradient feature extractors. To circumvent this illumination-related issue, several researchers tackled the subject and propose several solutions. In a global manner, color-space transformations are performed on the image. The main techniques are the Hue Saturation Lightness (HSL), Lightness and A and B (LAB), the Luma component (Y), and the Blue-difference and the Red-difference Chroma Components (YCbCr). The general assumption made for these color manipulations is that hue information does not change in the shadowed region of the image, implying that the hue information is not affected by the level of illumination of the image, or that the effects can be compensated. With a color extraction procedure, Cheng et al. [53] was able to remove moving vehicles and their light reflections on the image to extract the lane marks on the road. Katramados et al. [54] worked on color extraction and texture information with a temporal analysis to eliminate map lighting and water artifacts, including shadows, reflections and water prints. Álvarez et al. [55] proposed a segmentation using a grey–scale illuminant invariant image, which is computable in real time using a color camera, to find the road area.

The second category of image pre-processing techniques includes the pruning of non-relevant or misleading parts of the input sensor that can miss-lead the lane detection process. The apparent difficulty of the methods that fall into this category is to insulate the artifacts in the image sensor. To archive this objective, many studies have been developed over the years. In that context, objects like cars and pedestrians are treated like obstacles for lane detection. Therefore, several techniques have followed in order to detect them and remove them. Huang et al. [52] enhanced the detection process with 3D data from lidar. The lidar point clouds facilitate rapid removal and rejection of off-ground points that are considered as obstacles. One can notice that differentiation between obstacles is not made. A similar approach was presented by Hernández and Marcotegui [56] who were able to segment static or mobile elements from buildings and grounds using the 3D point cloud of a lidar transformed into a range image. Li et al. [57] used also the lidar’s point clouds to detect the free space in front of the vehicle, and then doing the lane detection in this free space. Using 2D flow of image points, Yamaguchi et al. [58] proposed a method based on the alignment of two successive images. The Structure-From-Motion technique was applied to infer the road region depending on the image motion. However, these techniques were later abandoned due to high false-positive rate [59].

The most utilized approach for the pruning part consists in defining some Region Of Interests (ROI). Hence, only these regions will be focused on the feature extraction. Several works have addressed the issue of how to determine these ROI. The most simple technique consists of taking the lower half of the image as ROI [60]. The naivety of these techniques makes it very limited. Therefore, other researchers use the correlation between the 3D world model and 2D image in order to delineate the ROI. To achieve this goal, it is required to know the camera pose with respect to the ground surface. Huang et al. [52] claimed that the pose is constant and hence the calibration was made beforehand. The same idea is shared by Nava et al. [61]. The authors proposed a ROI delimitation using the vanishing point of the road. But, as claimed by the authors, this method does hold only for small roll angle conditions. However, this strong assumption does not hold for curved roads in the case of two-wheeled vehicles. Therefore, Aufrere et al. [62] proposed a probabilistic road model that links the geometry of the road and the uncertainties about the camera pose. By doing so, the uncertainties about the model defined the ROI in the image. This idea was extended in [63]. Following, Kasmi et al. [64] proposed a top-down approach that first computes focused ROI using coarse maps such as OSM, then uses these ROI to perform coherent line detection.

The depth computation on an image can be used to determine the vanishing point on the image and hereby the delimitation of the ROI. Pomerleau [65] used a camera in front of a vehicle to extract the curvature of the lanes based on the intensity of the pixels, and then detected the vanishing point. The position of the vehicle into these lanes is also extracted with the pixel’s intensities. Zhang et al. [60] used also the pixel’s intensities into an image to detect the lanes and roads by segmenting the image. The segments are then divided into two classes: road and non-road. The vanishing point is then detected by extracting the edges of the road segments through a Hough transform.

As claimed before, feedback connections do exist between the higher functional blocks and the lower blocks. To illustrate that, an interesting approach is presented by Alvarez et al. [66]. It relies on the use of road priors and contextual information coming from a digital map in order to determine the shape of the road in the image. Depending on the number of lanes and the width of the lane, the road skeleton is built and smoothed using cubic interpolation. The retrieved road skeleton is then projected onto the bird’s eye view image by taking into account uncertainties related to the vehicle’s pose. Contrary to the previous methods, Cáceres Hernández et al. [67] emphasized on the collision risk region, which is extracted taking into account the vehicle speed, and therefore, the ROI size increases as the speed increases and vice versa.

#### 3.1.2. Feature Extraction

Once the irrelevant parts of the input sensor are discarded, the remaining relevant part of the input sensor is supposed to contain pieces of the lane marking. These pieces gathered together should contain all the necessary information needed in order to fit the lane marking. Throughout this work, these pieces are often called primitives or features. Indeed, feature extraction is a crucial step of lane marking recognition. Hence, in the majority of the works that fall in the model-driven approach, the fitting procedure is tributary of the outputs of the feature extraction. Therefore, in case of a momentary failure from the feature extraction module, recovery becomes almost impossible.

The study of this corpus of literature reveals that the majority of the approaches presented rely on bottom-up feature extractions. Indeed, lane markings are easily discriminated by shape and color in the image, and it is possible to determine whether a lidar beam has intercepted asphalt or road painting regardless of the lighting conditions [68]. Lane marks can be detected either based on their shape, color, or their combination [49]. To that end, several strategies have sprung up. The main assumption made is that lane markings are distinguished by their appearance from the rest of the road surface. This assumption leads to a whole family of filters based on gradient detection [62,69,70,71].

Another branch of solutions is to filter the edges that are not in the vertical direction. These filters are known as steerable filters [44]. Steerable filters enable to follow the orientation’s change along the lanes marking in the image by convolution with only three kernels. Following the same spirit, several hand-crafted filters were proposed to extract fragments of lane marking, namely, Sobel filters [72], Statistical Hough Transform [73], top-hat filter [74], and histogram-based filter [75]. A comparison of these features extractors have been presented by Veit et al. [76], the authors applied different feature extractions on the same input. Regardless of the type of gradient filter used, the kernel of the filter has to be adjusted before applying it to the image. However, the perspective distortion of the camera makes these adjustments not suitable for the entire image. To bypass this problem, a common approach is to transform the image into another perspective called the “inverse-perspective” image, also called the bird’s-eye view, as done in [44,70,77,78,79]. In the bird’s-eye view, the width of the lane markings is equidistant. This property has a lot of advantages, as for instance it is a convenient common space to fuse multiple sensor’s information. The representation facilitates some fitting produce that will be discussed below. However, these advantages come with a cost in terms of computational time and loss of resolution. When dealing with lidar data, the main assumption made is that lane marking can be distinguished based on their reflectivity. Therefore, authors in [80] presented a threshold in order to discriminate lane markings. This approach was extended in the work of Hata and Wolf [68]. The authors presented a thresholding method that separates the lidar point clouds into asphalt and road marking.

#### 3.1.3. Fitting Procedure

The main objective of the fitting procedure is to extract high-level representations of the path. This high-level representation is the sine qua non to a higher block of autonomous vehicles like decision-making and control. Thereby, the choice of the type of lane model is crucial. In practice, when fitting a model to noisy data there exists a compromise between over-restrained models that do not tolerate all the existing geometries and under-restrained models that tend to over-fit on noisy features.

Going down the timeline, preliminary works on lane detection focused on highway scenarios where the curvature’s change is small enough to be neglected. In that context, pioneering work on the road model was initially proposed by Dickmanns and Zapp [81] in 1986. The latter presumed building a mathematical 2D model that describes the lane geometries, yielding to a high-level representation of the lane marking with the use of a clothoid road model for planar roads. Dickmanns [82] extended the approach by using a 3D model lane representation that included a clothoid parameter and a curvature in the vertical direction. These works lay the foundations of several works with the objective to know what kind of lane model can represent accurately the lane marking and which fitting strategies should be adopted consequently.

Scrutinizing this body of the literature, the lane models can be clustered into three heterogeneous modeling techniques, namely parametric, semi-parametric, and non-parametric:

- Parametric model: Methods that fall into this category make the strong assumption of a global lane shape (e.g., lines, curves, parabola). These models tend to fail when dealing with non-linear road and lane topologies (merging, splitting, and ending lanes). Indeed, the geometric restrictions imposed by the parametric model does not tolerate such scenarios. Concerning the fitting strategies, several regression techniques have been used (e.g., RANSAC, least-squares optimization, Hough transform, Kalman filter)
- Semi-parametric model: Contrary to the parametric model, semi-parametric models do not assume a specific global geometry of the road. On the downside, the fitting model can over-fit or have unrealistic path curvature. The lane marking is parametrized by several control points. Different spline models with different control points have been used (e.g., Spline, B-spline, Cubic spline). The appearing complicatedness of these models is in choosing the best control points. Indeed, the number of these points affects the curve complexity. In addition to that, these points should be homogeneously distributed along the curve of the lane marking in order to prevent unrealistic curves.
- Non-parametric model: These models are the less conventional approach. The main needed prerequisite is continuous but not necessary differentiable. This model has more freedom to model the lane marking. Meanwhile, it is more prone to erroneous modeling, leading to unrealistic curves.

Such a classification allows a clear understanding of the distinctiveness of certain techniques. Furthermore, the assumptions made for each category help to understand the failures of some of these techniques. In the following, we propose in Table 3 a categorization of works regarding the aforementioned classification. We provide a summary of all the different categories presented and classify them according to their geometric methods, their fitting methods and their advantages or disadvantages. Although there are numerous modeling techniques for lane detection, none of them in able to correctly model lanes in all the cases encountered. The choice of a model is a trade-off between the complexity of the road scenarios and the complexity of the chosen lane model.

#### 3.1.4. Tracking Procedure

The vast majority of lane marking detection system integrates tracking mechanics that use knowledge from the previous frame to improve the knowledge on the present frame. According to Hillel et al. [49], this mechanics has three major goals: improving the accuracy of correct detection, reducing the required computation, and correcting erroneous detections. A tracking procedure can be used in a lane marking detection system in two different modes, that are using the detection results from the previous frame, or using tracking systems that enable the definition of ROIs in the current frame. By doing so, it will reduce the size of the ROIs. Aufrere et al. [62] used the vehicle’s information in order to update a probabilistic model, which contains all the ROIs delimitations. To do so, authors integrated these proprioceptive information in an Extended Kalman Filter. The same idea was adopted by Wu et al. [63]. These approaches can successfully decrease the signal-to-noise ratio by updating the ROI after each iteration. However, it should be noted that the main assumption made is that the model is capable of capturing the motion between two consecutive frames. At the same time, they assume that the latest detected lane marking is correct. Hence, no recovery strategy is made. Alternative approaches consist of fusing the lane marking detections and a digital map of the road that contains the position of the left and right lanes.

### 3.2. Learning Approaches

Historically, the concept of deep learning was founded in 1943 by Walter Pitts and Warren McCulloch [89]. However, it was not until the 2010s with the development of powerful computing machines with the arrival of Graphics Processing Units (GPUs), combined with the availability of the “big data” that was needed to train the models, that deep learning techniques became popular. The scope of applications of such methods extends to finance [90], healthcare [91], and self-driving vehicles [92].

One of the major challenges in estimating the lane marking is the need to have an accurate model that fits the detected features. Unfortunately, providing an accurate model that covers every road scenario is a complex task due to the singularity of some road scenarios (i.e., merging, splitting, and ending lanes). Moreover, inherent uncertainties coming from sensor data cannot be mathematically modeled in the model-driven pipeline system. Along these lines, monolithic end-to-end learning approaches have the advantage to abstract the mathematical modeling for each functional block, as presented in Figure 2. As a consequence, learning approaches, if treated properly, are a powerful tool to correctly detect lane markings.

On that subject, several studies have been deployed. In their study, Kim [87] reviewed the performances of classical machine learning methods for features extraction, namely, Artificial Neural Networks, Naive Bayesian Classifiers, and Support Vector Machines. Nevertheless, the fitting model was still performed using the Spline model. On the same topic, Gopalan et al. [93] introduced a learning-based approach based on a boosting algorithm to detect lane markings without requiring a predefined road model. The algorithm was validated on several data collected on daytime and nighttime, proving that the classical machine learning methods can be useful for lane detection marking.

Going back in time, the Convolutional Neural Network (CNN) gained notorious popularity in 2012 when Krizhevsky et al. [94] proposed AlexNet and won the ImageNet Large-Scale Visual Recognition Challenge (ILSVRC). Following the success of CNN methods in computer science, researchers considered its uses to tackle lane detection. In that regard, Huval et al. [95] were the first to use deep learning technique to identify pixel locations of the single lane on highways. Their model is based on OverFeat, a CNN developed by the team of Sermanet et al. [96]. They trained their CNN on a private collected dataset on a highway in San Francisco (USA). The ground truth labels were generated using a camera, Lidar, Radar, GPS, and human annotations. The network showed successful results in terms of lane detection. However, the CNN was restricted to the detection of only the ego-lane on which the vehicle was traveling. Indeed, the general assumption made is that the vehicle is always traveling in the center of the lane. Building on this success, He et al. [97] proposed a Dual-View Convolutional Neural Network framework for lane detection. The bird’s eye view images and the output of the network give line probabilities, which are fed to an optimizer in order to find the right lane marking. The network shows promising results but requires image pre-processing and post-processing.

Moreover, companies showed interest in this problematic. In that regard, researchers from Ford released an end-to-end framework called DeepLanes [98]. Unlike most of the works, the network detected lanes based on images coming from two laterally-mounted cameras looking downward onto the lane markers. Despite the good results, the network was not widely adopted due to the fact that the network was trained on a private database. Reproducibility was the watchword on the lane detection challenge which was held in CVPR’17 and in which the Tusimple database was released. The winner of the challenge was Pan et al. [99] with the Spatial Convolutional Neural Network, a deep learning technique designed for lane marking detection in traffic scene. The structure of this network allows it to exploit the spatial information in the image. On the same topic, Li et al. [100] proposed a network called Line-CNN. The network had slightly better results than the Spatial Convolutional Neural Network. The authors adapted the strategy of the regional proposal network, which exists in Yolo [101] for instance, to the lane marking detection. They also trained their network with an additional dataset which was not publicly released. A succession of neural networks has been released after that. However, as pointed out by authors in [102], many of the published works on deep learning for lane marking detection do not share their code, thus hindering the comparison.

The detection of lane markings using deep learning is an ongoing research topic, with multiple networks released every year dealing with the subject. The majority of these algorithms are benchmarked. Currently, the Tusimple benchmark, of which an excerpt is shown in Table 4, is saturated with high values in accuracy and F1 score. This can be explained by the fact that the dataset is not complex and the metrics are permissive [102]. In that regard, a database called CULane was released. The objective of this database is to offer a more complex and larger public dataset for lane detection. Considering the abundance of literature on the subject, tracking every deep learning algorithm proposed for lane detection is beyond the scope of this survey. However, a database named paperswithcode https://paperswithcode.com/ (accessed on 1 November 2021) reviews all the lane marking detection methods that has been benchmarked on either Tusimple or CULane. In Table 4 and Table 5, we provide an overview of the five best performing methods with available code on each benchmark, at the time of writing of this manuscript. Compared to the available tables on paperswithcode, we added whether extra training data were required in the CULane benchmark, and added the reference [103]. The difference between the top algorithms in both tables is only by a few percentages, whether it is based on the accuracy score in the Tusimple benchmark, or based on the F1 score in both benchmark. This means all of these algorithms are equivalent in terms of performance for lane detection.

### 3.3. Conclusion of Ego-Lane Level Localization

This section discussed the major algorithms for ELL. We emphasized methods that are based on the detection of the ego-lane markings. Accordingly, model-based approaches have a strong ability to detect the ego-lane marking in various scenarios. The sequential pipeline of these methods allows a better partition of the ego-lane marking detection task into blocks, where each one is responsible for a specific task. Therefore, the formalization of the whole problematic is enhanced, and the intermediate block representations allows a better system failure identification. Furthermore, such methods possess a systematic modular architecture that enables them to improve or incorporate new functionalities that were not supported in the initial design without requiring significant modifications. Regardless of the method used, a model is required to fit the detected features to a predefined road model. As a consequence, the generalization of these methods is complicated and challenging for highly complex road scenarios. As claimed by Hillel et al. [49], the model-based methods does perform well in the majority of road scenarios. On the other side, the monolithic learning approach based on Neural Networks reaches a better accuracy for the detection of lane marking as the majority of benchmarks’ leaders are deep learning methods, as shown in Table 4 and Table 5. Besides, they perform well when a model cannot be formalized or is not available. The learning approach requires a learning phase that is performed on an annotated dataset. The procedure is performed offline, preliminary to the deployment of the network. However, in real-world applications, data is limited in quantity and is usually gathered for a specific task and for a specific configuration (e.g., same city, same camera). Therefore, a change between the dataset and the validation test leads to a decline in the accuracy obtained. In addition, the learning procedure is time-consuming, and the output can not be predicted in advance. The major drawback of the methods based on this paradigm is that the network-based algorithm is hard to interpret as it is represented as “black boxes” to the user, which does not reveal why a certain error has occurred [48].

In the next section, we will discuss the last component of the algorithm localization, which is the Lane-Level Localization (LLL). A review of the literature is given, presenting the works and the techniques used to this end.

## 4. Lane-Level Localization (LLL)

A fundamental aspect of a fully autonomous vehicle is its ability to properly evaluate the situation of the ego-vehicle in regard to the road environment. Part of this situation evaluation is the knowledge about some key localization level components. In previous sections, we presented two components needed to fully fit this fundamental evaluation: the road level localization, or the knowledge of which road the vehicle is currently traveling on, and the ego-lane level localization, namely the knowledge of the position of the ego-vehicle in its lane. For the majority of the ADAS applications, a partial understanding of the observed road scene is enough, and therefore the knowledge of these two components is sufficient for the system to behave appropriately. However, the road understanding demands in terms of precision and false alarm rate [49] vary from one application to another. Therefore, for some tactical higher-level intelligent safety systems, the knowledge of the lane on which the vehicle is traveling is critical. Indeed, knowing the host lane can serve the autonomous navigation system in providing the most adequate instructions depending on this lane. For instance, such information can provide a better overtaking strategy.

In the broadest sense, Lane-Level localization is a meaningful concept that can refer to two distinct topics. The first goal is to determine the ego-lane, namely the lane on which the vehicle is currently traveling. Secondly, it may refer to the estimation of the lateral position of the vehicle inside the overall road. While the latter definition is an estimation problem of a real variable, the former one can be interpreted as a classification problem. The two paradigms lead to the same knowledge, which is the LLL or the localization of the host lane. There exist abundant systems that can help the autonomous vehicles to obtain the LLL. Some systems are using a GNSS receiver to locate the ego-vehicle in the road. The lack of accuracy provided by a classic GNSS, which can be caused by poor satellite signals, high dilution degree of precision, or multi-path in urban scenes, is first compensated with proprioceptive sensors such as the Inertial Measurement Unit (IMU). These methods are known as Dead Reckoning [111]. The position of the host lane is inferred by combining the obtained localization with a coarse digital map. Unfortunately, the accuracy of the localization obtained is in the order of several meters. Indeed, according to the Federal Aviation Administration [5], the accuracy of a standard GPS device is within 3 m with a 95% confidence, which cannot be sufficient for some ADAS that require a more precise localization. Moreover, when a road has multiple lanes, the problem becomes more complicated. Indeed, the localization obtained from the Dead Reckoning technique is not enough to precisely infer on what lane the vehicle is traveling. Hence, further information are used in order to refine this knowledge. This information can be produced by visual sensors or digital maps.

In practice, the localization of an autonomous vehicle can be performed by locating the vehicle with respect to some visual features, such as lane markings or traffic signs. These visual landmarks can either be detected using on-board sensors, or be already stored in digital maps. Concerning the Lane-Level Localization (LLL), the current literature abounds in solutions that address this issue in a variety of manners. However, two techniques stand out as the most fitting for this task. The first approach depends on very precise maps: these High Definition (HD) maps store the accurate position of landmarks (e.g., lane marking). As such, the system has to match the detected landmarks with the one stored in the maps. The second technique depends solely on the detection of visual landmarks such as lane marking with on-board sensors. For instance, lane marking systems are used to detect all the lanes on the road. Nevertheless, it is sometimes not possible to detect all the lane markings on the road due to occlusions from other vehicles. In the following, we present the state-of-the-art methods that are classified into two categories, that are the map aided approaches and the landmark approaches.

### 4.1. Map Aided Approaches

To reduce sensor dependencies, digital maps can store contextual information about the road. The amount of information stored depends on the scale of accuracy and detail displayed on the road network. In that sense, Du and Barth [112] described three scales of maps commonly used for autonomous vehicle systems, that are:

- Macroscale maps represent the road network with a metric accuracy. These maps are used for route-planning problems and high guidance routines. They provide the user meta information such as speed limitations or the number of lanes present on a given road. The road network is smoothed using clothoid curves, which can give a general intuition of the shape of the road.
- Microscale maps correspond to the most accurate maps. These maps have centimeters accuracy, representing the road network with dense information. Generally, lidars are used to gather maximum information. The fundamental benefit of these maps, which is their great information richness, is also their biggest disadvantage. Indeed, the density of information makes the handling of these maps difficult while trying to isolate points of interest, and keeping them updated is a laborious task.
- Mesoscale maps are a trade-off between the two aforementioned types of map. McMaster and Shea [113] claimed that a map has to provide enough details about the environment without cluttering up the user with unneeded information. As such, this kind of map has more accurate information compared to macroscale maps while not burdening itself with precise information as done by the microscale maps.

In the context of intelligent transport systems, mesoscale maps are the most used (e.g., Lanelets maps [114]) since they are the most suitable scale for intelligent vehicles, as it carries accurate information without being too dense. In addition, mesoscale maps have the merit of being easier to maintain compared to microscale maps. Therefore, a strong effort is currently made by the map providers to meet these requirements.

In recent decades, several researchers have deeply investigated the idea of using cameras and HD maps to have a successful and precise localization algorithm [115,116,117]. Generally speaking, the vision-based map matching localization is a process that aligns the perceived environment landmarks, such as lane lines, with the stored landmarks in the map. In this context, Li et al. [118,119] presented a lane Map-Matching algorithm using a mesoscale map with lane-level accuracy. The lane Map-Matching method is based on Multiple Hypothesis Technique, and no external sensor has been used to perform the Map-Matching. The experimental results were conducted in terms of lane Map-Matching accuracy. Within the same paradigm but in a different manner, Kang et al. [120] proposed a lane map-based algorithm for lane-level localization using a mounted camera on an autonomous vehicle. The method relies on the detection of lane markings in the image frame. The detected lane marking was matched using the GPS trajectory with the map database that contains the center-line of each lane. The map matching method was based on an Iterative Closest Point (ICP) based rigid map. The results showed an improvement in the accuracy in terms of localization. Indeed, the average error obtained by the state-of-the-art devices and GPS was 2.340 m, and this error was reduced to 0.475 m.

In the same manner, Ghallabi et al. [121] presented an approach in which a lane-level localization is performed using mesoscale map that is composed of many links (lane markings) represented by polylines. The objective of their work is to match a polyline (i.e., lane marking) detected with an onboard sensor to the corresponding lane markings stored in the map. To perform such a task, a lidar sensor is used to detect the lane marking. Afterward, the detected lane markings are matched with the corresponding map. The used map-marking strategy relies on a Particle Filter algorithm. In terms of evaluation, several experiments have been conducted and a cross-track metric has been used to evaluate the accuracy of the matching strategies. The results represented the error between the matched lane marking in the map and the ground truth lane marking stored in the map. Finally, the authors came to the conclusion that their current framework is promising and sufficient for highway use-cases. However, the authors did not explicitly explain how the errors in lane marking position will affect the ego-vehicle localization. In addition, the errors that can be stored in the map are not taken into account, which can also affect the accuracy obtained as pointed out by Welte et al. [122].

Nevertheless, the majority of the lane-based Map-Matching techniques only consider the ego-lane marking lane in order to perform the map-matching. To overcome this issue, Suhr et al. [123] propose to include all the lane markings in the lane-based-Map-Matching algorithms in which a Particle Filter (PF) is used. The performance of the general solution is considerably stable even in urban crowed scenarios. Indeed, the experiments showed that the average lateral error is about 0.50 m which is less than the width lane (3 m in Korea) which led the author to conclude that it is sufficient to recognize driving lanes. In the same spirit, Cui et al. [124] proposed to incorporate the lane-type information to the lane-based-Map-Matching algorithm with the type of lane marking. These techniques claim a lane-level localization. However, none of these techniques consider the ego-lane index for map matching which is questionable if we take into consideration that in some cases, typically highway scenarios with multi-lanes, ambiguities may exist in distinguishing the true lane. Indeed, all the lane marking shapes are identical, which results in increasing the difficulty for choosing the correct lane since there exists a strong ambiguity on choosing the right lane.

To address this issue, Lee et al. [125] proposed an atypical approach, using a sequential framework that is composed of two sequential deep learning blocks. The first deep learning block detects all the lane marking present in the image. The second block is a Long Short-Term Memory (LSTM) network that identifies and remembers the lane on which the vehicle is traveling. Finally, the lane information, which is the output of the latter block, is used in a lane-based Map-Matching.

### 4.2. Landmark Approaches

In these approaches, relevant road level features are extracted from images. Once these features are extracted, they are fed into a high-level fusion framework that assesses the number of lanes and on which one the vehicle is travelling. In that context, Lu et al. [126] estimated the probability of belonging to a lane using lane change information and lane-marking detectors. In the quest of LLL, Nieto et al. [71] presented a LLL based on multiple-lanes detection. First, an ego-lane detection is performed to detect the ego-lane marking and the lane geometry (curvature and lane width). Afterward, based on the estimation of the vanishing point, reconstruction of the geometry of the road is estimated, which gave an indication of the number of lanes. In addition, an assumption on the geometry of the road is made. This assumption formulates that the lanes on the same road share the same curvature and the same lane’s width. Taking into account these considerations, adjacent lanes are hypothesized and tested. The verification of these hypotheses is performed by a confidence level analysis, which is based on distance measurement.

Popescu et al. [127], Popescu et al. [128] presented a probabilistic formulation of the problematic, first on intersection [127], and then on more general road situations [128]. The lane marking information, together with some relevant visual landmarks like arrows in the lane, are fused in a semi-fixed Bayesian Network. The main objective of this network is to estimate instantaneous probabilities for each lane position hypothesis. The semi-fixed structure of this Bayesian Network allows the addition of more observations, such as adjacent lane marking and more arrows in the road. Eventually, the experimental results showed promising results in identifying the ego-lane. In the same manner, Ballardini et al. [129] proposed a LLL method based on a Hidden Markov Model (HMM) to filter the outcome of a marking-lane detector based on stereo images. To do so, the HMM is modeled based on a lane detector, for which some score functions are introduced depending on the reliability of the detector. Results on real datasets show very good results. Nevertheless, lane-changing situations have not been addressed. In addition, the probabilistic HMM calculation and formalization were not explicitly defined, leading to a non-intuitive definition for the emission and transition probabilities. Kasmi et al. [130] proposed an improvement of the latter work by defining a formalization of the HMM that avoids empirical definitions and thus leads to a better understanding of the system. Furthermore, they use the knowledge of surrounding vehicles to better infer the correct number of lanes. Following this idea, a recent approach presented by Volvo researchers Svensson and Sörstedt [131] takes advantage of the surrounding vehicle. The method is based on a Bayesian Filter that fuses the position of surrounding vehicles detected, a map that provides the lanes number, and an ego-lane marking that gives the ego-lane geometry. The objective of the Bayesian filter is to infer the position of the ego-lane. The early tests show promising results. However, the main drawback of this technique is that it is tributary to the presence of vehicles. Furthermore, real-world experimental results are missing to assess the efficiency of the approach, especially when there is no surrounding vehicle.

### 4.3. Conclusion of Lane-Level Localization

In this section, literature about Lane-Level Localization has been studied and most relevant works have been presented. In the broadest sense, Lane-Level localization is a meaningful concept that can be related to two different problematics. The two paradigms lead to the same knowledge, that is the position of the vehicle on the road, but differ in the methodology.

The first is the knowledge of the lateral position of the autonomous vehicle with respect to the road. The solutions of this problem yield a lateral position that is a real number, and are usually computed using a map-aided approach. In this paradigm, lane-level Map-Matching algorithms are used to match the estimated position of an ego-vehicle, which can be estimated using Bayesian filters (e.g., Kalman filter) with the proprioceptive sensors. This estimated position is then matched with a map. Generally speaking, the type of map used for this kind of task is the mesoscale map [118,119] using a lane-level Map-Matching algorithm. Contrary to the Map-Matching methods presented in Section 2, this kind of algorithms faces more difficulties in ambiguous cases. Typically, for highway scenarios with multi-lanes, strong ambiguities exist as all the lane marking shapes are identical. The second limitation of such a paradigm is in the type of map used. Indeed, these maps are relatively complex to build and cost-intensive, in addition to being difficult to use as they are oftentimes not open-source.

The second paradigm uses a different methodology in order to solve the LLL problematic. The methods that belong to this group of paradigm articulate the knowledge of LLL as a classification problem. To do so, these methods rely on the relevant features that are present in the road scene, especially lane markings and adjacent vehicles. These relevant features are first detected and then fused in high-level fusion frameworks that are essentially based on a graphical probabilistic model, namely, Bayesian Network [127] or Hidden Markov Model [129]. These probabilistic frameworks have the ability to take into consideration uncertainties of the detected relevant features. Contrary to the first paradigm, these methods rely solely on the exteroceptive sensors that are embedded in most of autonomous cars. Furthermore, they do not use expensive maps and thus are more flexible.

## 5. Overall Conclusions

In this survey, we presented a taxonomy of the state-of-the-art methods for localization of autonomous vehicles on highways. The task of localization is split into three components that are the Road Level Localization (RLL), Ego-Lane Level Localization (ELL) and Lane-Level Localization (LLL). The Road Level Localization part aims at finding on which road the vehicle is currently traveling on. Techniques to perform such a task are named Map-Matching methods and can be divided into two categories that are the deterministic and probabilistic models. Without surprise, deterministic models offer lower computational demands at the cost of being less accurate than their probabilistic counterparts. Indeed, the probabilistic methods can keep multiple hypothesis or take into account the temporal dependencies of the estimation (e.g., the vehicle cannot switch roads between two timestamps), leading to more solid frameworks. Once the vehicle is able to pinpoint itself in a map, its second goal is to locate itself in its lane. This task is called the Ego-Lane Level Localization (ELL), namely the task of localizing oneself relatively to the ego lane. Two main approaches exist to tackle this problem, that are the model approach and the learning approach. In the first one, the estimation is conducted by splitting it into submodules that pre-process the sensors data, extract features, fit them to lane markings and finally track the detections between the frames. This approach allows good failure detection, as each block is simple enough to supervise them. In the learning approach, a neural network is trained on road data to be able to directly extract the lane markings (and thus the robot position relatively to them). The inherent nature of the neural networks allows to better take into account the context of the scene, thus reaching better results than model approaches. However, by this same nature, learning approaches suffer from their little explainability, and require considerable training sets to contain all possible road scenarios. Finally, the last part named Lane-Level Localization (LLL) consists of finding on which way the vehicle is currently driving. Two options are possible, that are either locating the robot relatively to the overall road or apprehend the problem as a classification exercise to extract on which lane the robot is traveling. The first solution uses maps to aid it in the localization, but suffer from ambiguities in the case where several identical lanes are detected. The second solution chooses to classify each lane and selects the most likely among them. To do so, the methods take advantage of features extraction from the sensors data and from the adjacent vehicles. Furthermore, they have the benefit of not using maps that are costly to produce.

In our opinion, several research directions are worth exploring. First, the use HD maps is appealing because of the inherent resulting precision of localization. However, these maps are not easy to build or maintain. Indeed, they require high computational power and considerable amounts of storage, while their quality heavily depends on their update frequency. Thus, researches about collaborative construction and update of such maps can lead to a decreased cost as well as a better coverage around the world [132]. Second, deep learning techniques also prove themselves useful, despite a lack of guaranties and their appetite for expansive databases. As such, works aiming at reducing their data consumption and monitoring their outputs would lead to safer and more resilient systems [133]. Finally, the classical methods still have a role to play because of their simplicity and their detection guarantees. In a world where power consumption is becoming a fundamental consideration, small, resilient systems are as much attractive as complex systems, even at the cost of a slightly lower accuracy.

## Figures and Tables

**Table 1:** Performance of four Map-Matching methods, from [10]. The accuracy represents the percentage of correctly matched samples. Each interval depicts the worst-to-best performance range.

Method	Matching Accuracy
point-to-curve	53–67%
point-to-curve, considers heading	66–85%
point-to-curve, enforces route contiguity	66–85%
curve-to-curve	61–72%

**Table 2:** Summary of Map-Matching algorithms in terms of uncertainty-proof, matching break, integrity indicator and run time.

Methods	Uncertainty-Proof	Matching Break	Integrity Indicator	Run Time
Deterministic methods				
Geometric	−	−−	−−	++
Pattern-Based	−	−−	−−	++
Probabilistic methods				
Hidden Markov Model	+	0	+	+
Conditional Random Field	+	+	+	0
Particle Filter	+	−	++	+
Weighted Graph Technique	+	−−	+	+
Multiple Hypothesis Technique	+	+	++	−

**Table 3:** Classification of lane fitting models presented in the literature, dissected into three main categories that are the parametric, semi-parametric and non-parametric approaches.

Categories	Geometric Methods	Fitting Methods	Advantages	Disadvantages	References
Parametric	Straight lines	Hough transform and its variants	Straightforward approach shows good approximation for short range lane marking and can be valid in highway scenarios	Unfit for curves roads which is the cases in most rural roads	[60,65,77,79,83,84]
	Polynomial model	RANSAC, least squares optimization	The spectrum of application is greater than the linear model. In addition, Polynomial models has the ability to estimate the parameters of the road.	Can not handle abrupt change of curvature. The geometrical assumptions are not always correct (e.g., taking 3–3.5 m as a width lane)	[52,62]
	Cloithoid	Extended Kalman filter	Can handle situations where there is a abrupt change of the steering angle (e.g., at the junction of a straight and curved roads)	The clothoid model is generally made of some simplifications in order to get a viable model	[85,86]
Semi-parametric	Splines	Energy-based optimization	Capable of dealing with a large range of curved road using control points if accurately chosen	The inconvenience of this model appears in the choice of the control points. Undoubtedly, the position of these control points will affect the general curve of the lane. A wrong choice of theses control points leads to unrealistic road shape.	[58,87]
Non-parametric	Isolated points	Particle filter	The model is not governed by geometric restrains, which allows it to model more challenging road lane marking.	With no geometric restrains imposed, the fitted model can leads to unrealistic road model. Indeed, geometric correlations between lane marking are not considered.	[88]

**Table 4:** Excerpt from the best performing deep learning algorithms benchmarked in Tusimple in terms of accuracy and F1 score.

Models	Accuracy	F1 Score	Extra Training Data	Paper Title
RESA	96.82%	96.93%	No	RESA: Recurrent Feature-Shift Aggregator for Lane Detection [104]
PINet	96.75%	97.20%	No	Key points estimation and point instance segmentation approach for lane detection [103]
ENet-SAD	96.64%	95.92%	No	Learning lightweight lane detection cnns by self attention distillation [105]
HarD-SP	96.58%	96.38%	No	Towards Lightweight Lane Detection by Optimizing Spatial Embedding [106]
CondLaneNet	96.54%	97.24%	No	CondLaneNet: a Top-to-down Lane Detection Framework Based on Conditional Convolution [107]

**Table 5:** Excerpt from the best performing deep learning algorithms benchmarked in CULane in terms of F1 score. The results showed stands for the total of all classes of the CULane.

Models	F1 Score	Extra Training Data	Paper Title
CondLaneNet	79.48%	No	CondLaneNet: a Top-to-down Lane Detection Framework Based on Conditional Convolution [107]
LaneAF	77.41%	No	LaneAF: Robust Multi-Lane Detection with Affinity Fields [108]
SGNet	77.27%	No	Structure Guided Lane Detection [109]
LaneATT	77.02%	No	Keep your Eyes on the Lane: Attention-guided Lane Detection [110]
RESA	75.3%	No	RESA: Recurrent Feature-Shift Aggregator for Lane Detection [104]
